# Anomalous Origin of the Right Coronary Artery: An Uncommon Presentation

**DOI:** 10.7759/cureus.25494

**Published:** 2022-05-30

**Authors:** Mohammed Shaban, Pravash Budhathoki, Tanushree Bhatt, Somin Lee, Ana P Urena Neme, Miguel A Rodriguez Guerra, May Zaw

**Affiliations:** 1 Internal Medicine, BronxCare Hospital Center, Icahn School of Medicine at Mt. Sinai, New York, USA; 2 Cardiology, Medicina Cardiovascular Asociada, Santo Domingo, DOM; 3 Medicine, Montefiore Medical Center, Albert Einstein College of Medicine, Bronx, USA

**Keywords:** left sinus of valsalva, tachycardia induced cardiomyopathy, abnormal origin of right coronary, ectopic coronary, anomalous origin of coronary artery

## Abstract

The anomalous origin of the coronary artery is a relatively uncommon condition with a variant incidence depending on the modality of the imaging techniques such as transesophageal echocardiography (TEE), computed tomography angiography (CTA), magnetic resonance angiography (MRA), or invasive coronary angiography (ICA). The importance of diagnosing ectopic coronary artery origin comes from its possible relation to sudden cardiac death (SCD) cases in young populations. The anomalous origin of the coronary artery could cause myocardial ischemia and fibrosis; this would, in turn, increase the chances of fatal ventricular arrhythmias. In this report, we present a 40-year-old male, incidentally found to have persistent tachycardia and a gradually decreasing left ventricular ejection fraction (LVEF). He denied any symptoms or changes in his baseline, unlimited, functional capacity. However, his records were remarkable for persistent tachycardia over more than six months, raising concerns about tachyarrhythmia-induced cardiomyopathy related to his anatomical variations. We also discussed the guideline-directed therapeutic option for the abnormal origin of the coronary artery as per current guidelines.

## Introduction

An anomalous origin of the coronary artery is an uncommon condition (weighted prevalence is 0.26%) [1]. The origin of the left main coronary artery or left ascending artery (LAD) from the right sinus of Valsalva or right coronary artery (RCA) is referred to as anomalous aortic origin of a coronary artery (AAOCA) [1]. It can be grouped into interarterial (with or without an intramural course), prepulmonic, subpulmonic, retroaortic, and retrocardiac [1]. Complications rarely occur during or immediately after exercise, although sudden death may occur without prior symptoms. The underlying reason is that exercise can cause expansion of the aortic root and pulmonary trunk, which, in addition to external coronary artery compression, increases the pre-existing angulation of the coronary artery takeoff, decreasing the luminal diameter in the proximal portion of the coronary artery with subsequent myocardial ischemia [2]. This is a case of a patient who presented with asymptomatic tachyarrhythmia-induced cardiomyopathy associated with an anomalous origin of the RCA.

## Case presentation

**Figure 1:**
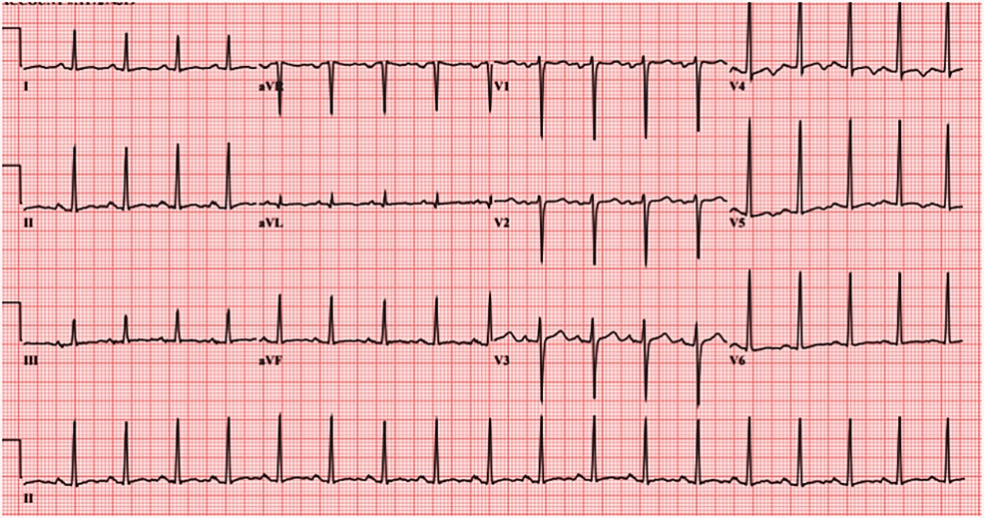
EKG shows sinus tachycardia and non-specific ST and non-specific T wave changes.

A 40-year-old male with unlimited functional capacity was sent to our institution after he was found to have a heart rate (HR) of 120 beats per minute (bpm) on the pre-employment screen. He reported that his HR had been 100 to 115 bpm during his follow-ups for chronic medical conditions. He has a history of type 2 diabetes mellitus, hypertension, and lisinopril-induced angioedema. Our patient received amlodipine 5 mg once daily, carvedilol 6.25 twice a day, metformin 1000 mg twice daily, insulin glargine 15 units at bedtime, and insulin apart five units daily. He has been consuming 1-2 beers and smoking one pack a day for the last four years. His vital signs were significant for tachycardia at 117 bpm. The urine drug screen came negative; the electrocardiogram (EKG) showed sinus tachycardia with non-specific ST and T wave changes (Figure 1); the chest radiograph showed no evidence of cardiopulmonary disease.

Through the medical record review, we found that six months ago, when he was admitted due to the angioedema, his transthoracic echocardiogram (TTE) showed concentric left ventricular hypertrophy (LVH) with global wall motion abnormality, mildly decreased left ventricular ejection fraction (LVEF) of 45%, grade 1 diastolic dysfunction, and mild to moderate pulmonary hypertension; he was discharged with an HR of 95 bpm. His HR was found between 102 and 110 bpm in another presentation with alcohol intoxication and hyperglycemia three months before the current admission. He was started on carvedilol 6.25 twice daily.

**Figure 2:**
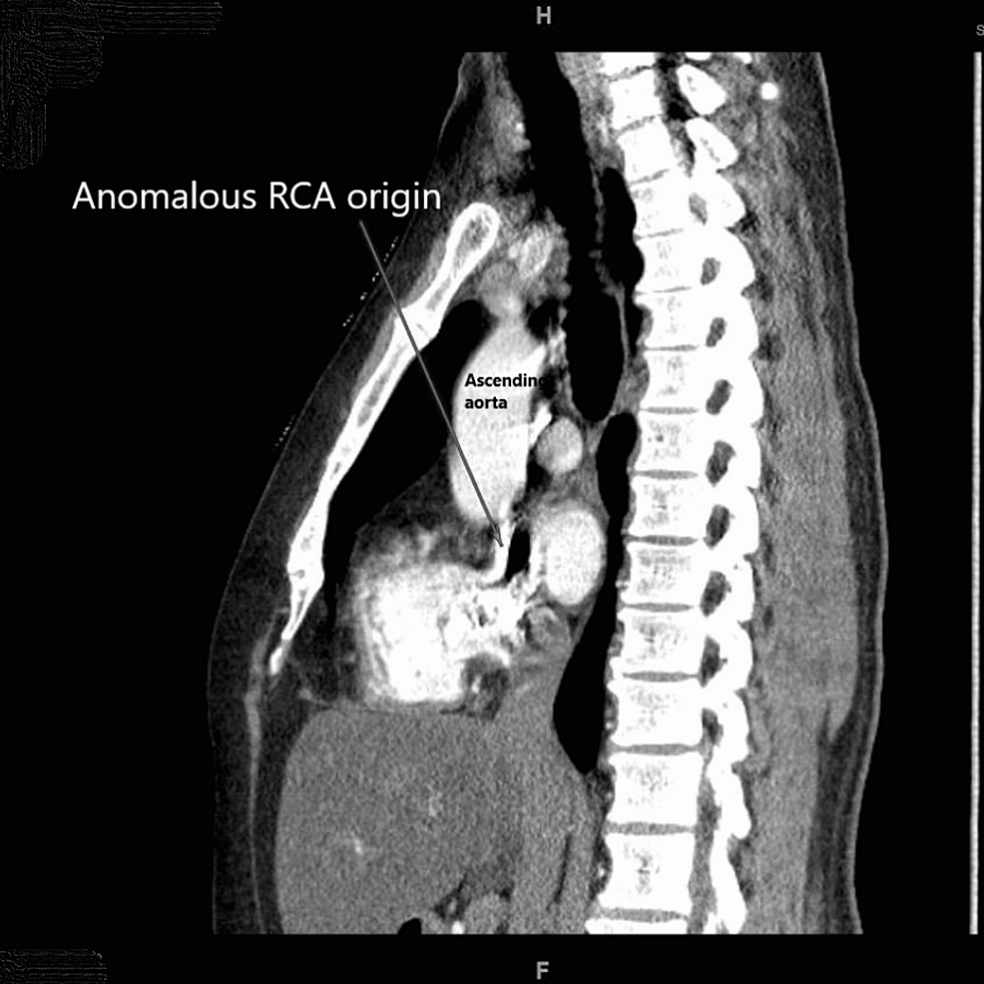
CTA chest shows an incidental anomalous origin of the right coronary artery from the left sinus of Valsalva (sagittal view).

**Figure 3:**
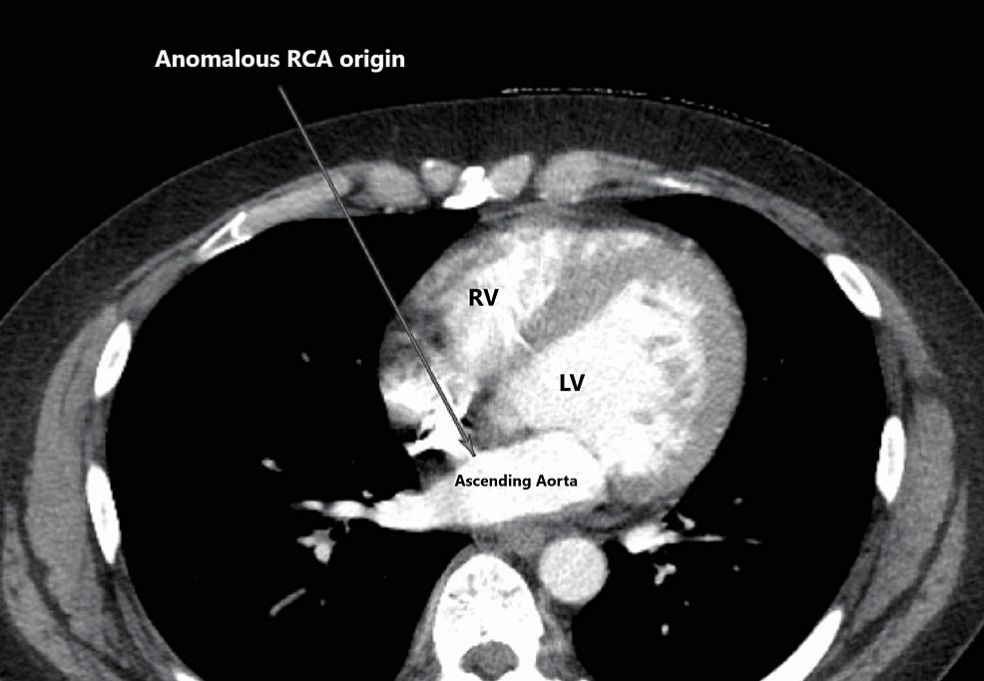
Anomalous origin of the right coronary artery from the left sinus of Valsalva.

A computed tomography (CT) pulmonary angiography was done to rule out a subclinical pulmonary embolism. It incidentally showed an anomalous origin of the right coronary artery from the left sinus of Valsalva (Figures 2-3).

The current TTE showed a progressive decline of the LVEF to 20% (compared to 45% three months ago) with a severely dilated left ventricle and global hypokinesis. Hydralazine 10 mg/Isosorbide dinitrate 10 mg was added to current anti-failure measures. Angiotensin receptor blockers (ARBs) were deferred given the history of lisinopril-induced angioedema complicated with acute respiratory failure six months ago. He was anxious about further modalities of cardiac imaging utilizing any contrast materials.

Our patient, who had no family history of cardiac or autoimmune disease, was asymptomatic with no clear etiology for the decremental decrease in LVEF. He was discharged with a plan to up-titrate anti-failure core measures and perform a CT coronary angiogram. At the later outpatient visit, he was asymptomatic with an HR of 110-117. Unfortunately, he is still uncomfortable undergoing any contrast-based imaging.

## Discussion

Multiple variants of anomalous origin of coronary arteries are present and categorized by the ectopic origin site of the coronary artery, which either arises from the aorta, a wrong sinus Valsalva, pulmonary artery, a branch of another coronary artery, or other arteries such as the brachiocephalic trunk, innominate, left mammary, left subclavian, carotid artery, or bronchial artery [3]. The ectopic origin site from the aorta, the anomalous aortic origin of coronary arteries, is further categorized into inter-arterial, subpulmonic, pre-pulmonic, retro-aortic, or retro-cardiac. These congenital artery anomalies could be malignant and contribute to sudden cardiac death (SCD) due to their abnormal courses and usual acute angles, such as in inter-arterial anomalies between the aorta and pulmonary arteries [4].

Different diagnostic tests are capable of revealing coronary artery abnormalities, including transesophageal echocardiography (TEE), computed tomography angiography (CTA), magnetic resonance angiography (MRA), and invasive coronary angiography (ICA) [1]. Historically, TEE was complementary to classic left heart catheterization to assess coronary artery anomalies [5]. The coronary artery's anomalous origin prevalence depends on diagnostic modality: 0.05-0.1% in ICA [6], 0.15% in Echo, 0.82-1% in CT angiography, and 0.70% in MRA [7]. The prevalence of inter-arterial anomalous left coronary artery (ALCA) was 0.03%, and inter-arterial anomalous right coronary artery (ARCA) was 0.23%. Inter-arterial ARCA is more common, but inter-arterial ALCA is more prevalent in SCD [1].

The most common anomalous origins of coronary artery populations are asymptomatic and benign. Still, these anomalies are associated with cardiac arrhythmia, heart failure, angina, myocardial ischemia, and SCD [8]. SCD is a tragic, detrimental event for their families and the public health of young people. The etiology of SCD was studied in many retrospective studies. SCD is associated with cardiomyopathies, channelopathies, myocarditis, ischemic heart disease, atherosclerotic coronary artery disease, anomalous origin of coronary arteries, aortic dissections, mitral valve prolapse, valvopathies, idiopathic left ventricular hypertrophy, non-ischemic left a ventricular scar, or structurally normal heart [9]. A meta-analysis by D’Ascenzi et al. [10] reported that the anomalous origin of coronary arteries accounts for 7.2% of the cause of SCD among athletes and 1.9% of SCD among non-athletes [10]. The anomalous origin of the RCA from the left coronary sinus accounts for 0.02% to 0.2% of anomalous coronary artery origins [11].

The anomalous aortic origin of the left or right coronary artery with a malignant inter-arterial course is associated with an increased risk of SCD [3]. The exact pathophysiology of SCD of anomalous origination of coronary artery from the opposite sinus (ACAOS) is unknown, but several mechanisms propose tissue ischemia as a cause. In other words, acute angle takeoff, knicking compression of coronary vessels, ostial abnormalities including ostial valve-like ridge, slit-like orifice, flute beak-shaped ostium, vessel segment spasm, compression of anomalous coronary artery intramurally or between the great arteries, and compressibility between the aorta and pulmonary artery could eventually cause reduction of coronary artery flow and lead to inadequate tissue perfusion and hypoxia [3]. These accumulative episodes of myocardial ischemia cause patchy myocardial necrosis and fibrosis and are likely the culprits for ventricular arrhythmias and sudden cardiac death [12]. During physical exercise, emotional burden, and tachycardia, myocardial oxygen demand increases while coronary flow decreases. This mismatch leads to cumulative myocardial ischemia [13] and fibrotic scar, eventual lethal ventricular arrhythmia, myocardial ischemia, and sudden cardiac death [14]. This theory was confirmed by the pathological feature of postmortem sudden cardiac death victims of anomalous origin coronary artery demonstrates histopathologic finding of left ventricular subendocardial myocardial fibrosis [15]. Our patient could be proof of this theory as well. He had a gradually decreasing LV systolic function, despite being asymptomatic over a few months. However, the ectopic origin was that of the RCA, which generally contributes less to LV blood supply than other coronary vessels.

The ACAOS population can also present with dyspnea, palpitation, syncope, angina, or without symptoms [16]. Our patient with an anomalous origin of the RCA from the left sinus of Valsalva has a non-specific perception of palpitation. The current TTE showed a progressive decline of the LVEF to 20% (compared to 45% three months ago) with a severely dilated left ventricle and global hypokinesis. Although our patient has a history of alcohol dependence, hypertension, and type 2 diabetes mellitus, we cannot completely rule out the possibility of an association between tachycardia-induced cardiomyopathy and his congenital anomalous coronary artery origin.

Tachyarrhythmia can lead to reversible cardiomyopathy caused by ventricular dilation and systolic dysfunction. The pathophysiology of tachycardia-induced cardiomyopathy is the depletion of ATP, decreased myocardial blood flow, and increased oxidative stress, causing loss of fibrillary function and leading to ventricular dysfunction [7]. Exaggerated ACE production in response to tachycardia increases angiotensin-2, causes myocyte elongation, left ventricular enlargement, and changes the wall stress [17].

The American College of Cardiology and American Heart Association guidelines suggest three treatment options for those who have symptomatic ACAOS: medical treatment, observation, coronary angioplasty with stent, and surgical repair [8]. Conservative medical/observation treatment includes beta-blockers and restriction of severe physical exertion. For surgical options, symptomatic patients with syncope, angina, ventricular arrhythmia, and evidence of ischemia on provocative testing are warranted [7]. In the case of inter-arterial anomalous aortic origin of the left coronary artery (AAOLCA), even if they are asymptomatic, it is often referred to for surgical intervention due to the increased risk of SCD with this anomaly. The surgical repair is called the "unroofing" procedure. It is the choice of procedure for inter-arterial, intramural anomalous aortic origin of a coronary artery (AAOCA) [8,18]. The operation is performed via an incision of the segment of the anomalous coronary artery and relocation to the appropriate sinus and re-implanting. Anomalous aortic origin of the right coronary artery (AAORCA) surgical interventions are less favorable and may not sometimes warrant surgical intervention [8,18]. Instead, stent-angioplasty of an obstructed intramural segment of AARCA is applicable in the presence of disabling symptoms and high risk for RCA, stenosis greater than 50%, large dependent myocardial regions, and documented reversible ischemia [8]. In our case, the plan is to perform a CT coronary angiogram to exclude any stenotic lesions once our asymptomatic patient agrees. Other therapeutic modalities have to be considered accordingly.

## Conclusions

Our patient is a unique case with an anomalous origin of the right coronary artery from the left sinus of Valsalva and is presented with a progressive decremental decrease of LVEF, probably associated with his persistent asymptomatic tachyarrhythmia. Further follow-up is still needed to determine the course of the disease or to establish if it is a reversible condition. Coronary angiograms, with or without invasive intervention, are still considered once our asymptomatic patient agrees. This phenomenon was not reported in the prior literature review, as per our research.
